# Cardiovascular magnetic resonance assessment in previously repaired ALCAPA

**DOI:** 10.1186/1532-429X-13-S1-P192

**Published:** 2011-02-02

**Authors:** Aurelio Secinaro, Hopewell Ntsinjana, Vivek Muthurangu, Oliver Tann, Marina L Hughes, Victor Tsang, Andrew M Taylor

**Affiliations:** 1 Great Ormond Street Hospital for Children - Institute of Child Health, London, UK

## Introduction

Anomalous origin of the left coronary artery from the pulmonary artery (ALCAPA) is a rare coronary artery anomaly.

Purpose: The aim of this study is to show the role of cardiovascular magnetic resonance (CMR) in assessing these patients.

## Methods

6 patients with repaired ALCAPA (2 Tackeuchi, 4 direct re-implantation) underwent CMR to assess clinical suspicion of myocardial ischemia - short axis and long axis cine images (assess ventricular function), late-gadolinium enhancement (detect myocardial fibrosis), adenosine stress perfusion scan (detection of reversible ischaemia) and 3D whole-heart imaging (visualization of proximal coronary arteries).

## Results

The LV function was preserved in all patients (mean LVEF= 62.7% ± 4.23%). The LV volumes were within the normal ranges LV (mean indexed LVEDV=75.4 ml ± 3.5, mean LVESV= 31.6 ml ± 9.37) In one patient, hypokinesia of the anterior segments was visualized. 5 of the 6 patients showed sub-endocardial late gadolinium enhancement involving the antero-lateral wall and the anterior papillary muscle. 4 of the 6 patients presented areas of inducible ischemia, reversible at rest. In 3 of the 6 patients the proximal course of the left coronary artery wasn't clearly visualized. In these patients, proximal the LCA obstruction was confirmed during invasive coronary angiography.

## Conclusions

CMR is a good, non-invasive, radiation-free investigation in the post-surgical evaluation of ALCAPA. We show that antero-lateral sub-endocardial myocardial fibrosis is a characteristic finding. Furthermore, stress adensoine CMR perfusion, can identify reversible ischaemia in this group. Such imaging may be a promising tool in the future clinical decision -making.

**Figure 1:**
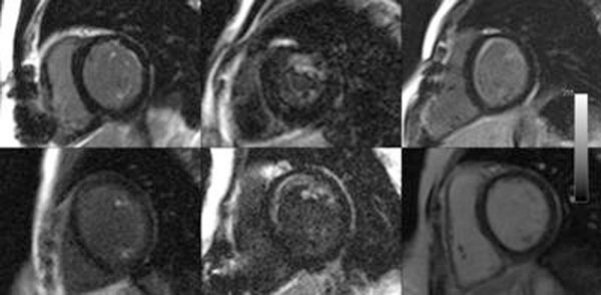
The charachteristic LGE in our repaired ALCAPA population .

**Figure 2:**
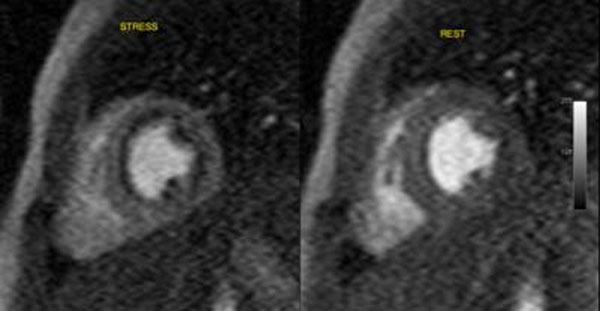
Perfusion defect in repaired ALCAPA.

**Figure 3:**
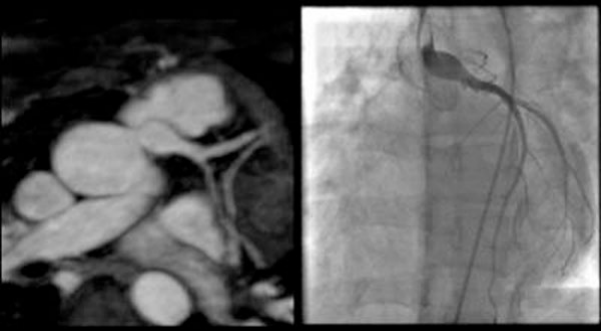
3D whole-heart imaging of ALCAPA repair compared with invasive coronary angiography(Tackeuchi technique)

